# Intolerance of uncertainty fuels depressive symptoms through rumination: Cross-sectional and longitudinal studies

**DOI:** 10.1371/journal.pone.0224865

**Published:** 2019-11-19

**Authors:** Vivian Huang, Mabel Yu, R. Nicholas Carleton, Shadi Beshai

**Affiliations:** 1 Department of Psychology, Ryerson University, Toronto, Ontario, Canada; 2 Department of Psychology, University of Regina, Regina, Saskatchewan, Canada; INSERM CEA Cognitive Neuroimaging Unit, FRANCE

## Abstract

The current study replicated and extended previous studies by examining the mediating and moderating role of rumination in the relationship between intolerance of uncertainty (IU) and depression in a community sample using both cross-sectional (*n* = 494; 56.9% female) and a two-months longitudinal (n = 321; 48.4% female) designs. Participants in each study were recruited through online crowdsourcing websites and completed study questionnaires. Results from Study 1 suggested that, while rumination did not appear to moderate the relationship between IU and depression, rumination appeared to partially mediates such relationship. Results from Study 2 supported rumination as fully mediating the relationship between IU and depression over two months. The brooding and reflection rumination subtypes exerted a significant indirect, but not moderating, effect on the relationship between IU and depression. Brooding exhibited a stronger mediation effect than did reflection. Overall, current results suggest that high levels of IU fuel the development of depression symptoms over time through engagement in heightened rumination. The IU-depression association appeared fully explained through rumination as it is a passive and contextually-dependent coping response that may enhance individuals’ emotion and facilitate the development of depressive symptoms.

## Introduction

Approximately 10–16% of people living in Western nations will experience clinical depression at some point in their life [1]. Depression is a multifaceted condition associated with several somatic, affective, cognitive, and social symptoms [2]. Depression is also a very burdensome condition and the leading cause of disability in the world [3]. Consequently, understanding the nature, mechanisms, and correlates of depression is vital, as such discoveries pave the way for refining existing treatments and designing novel treatments. Elucidating factors that make certain individuals more vulnerable to developing depression is also important for diagnosis and prevention. Understanding the interactive properties of vulnerability factors in depression is key, as such interactions systematically change the nature and presentation of the disorder. For example, new evidence suggests rumination and intolerance of uncertainty (IU) may indirectly affect depressive symptoms, acting as stable cognitive vulnerabilities for symptom onset and maintenance, and potentiating novel treatment target e.g., [4].

### Rumination and depression

Rumination has consistently been associated with depression and correlate constructs. Rumination is a style of thinking typified by repetitive and passive focusing on symptoms of distress, their causes, and their consequences [5]. Ruminative styles are also associated with repetitive thinking about past events, their meaning, and their consequences [6]. Rumination has generally been studied extensively in the context of depression, with compelling evidence that this thinking style is a vulnerability factor in depression onset and maintenance. For example, rumination has been positively correlated with depressive symptoms in cross-sectional studies [7], and has been linked with heightened depression symptoms in longitudinal studies [8]. Researchers have also used experimental designs to demonstrate the relationship between experimentally induced rumination styles and negative affect, negative thinking, and interpretation biases [9–11]. Problematically, using rumination as a strategy to regulate negative emotions tends to exacerbate distress [12]. Meta-analytic results suggest that rumination as an emotion regulation strategy is associated with anxiety and depression, as well as substantial symptom exacerbation [13]. Rumination may serve as an avoidant function, therein worsening depression symptoms through related strategies, such as emotional or behavioural avoidance [14,15]. Accordingly, rumination appears to be a maladaptive and paradoxical strategy for regulating negative emotions.

Researchers have identified two subtypes of rumination: reflection and brooding [16–19]. Reflection rumination is typified by attempts to understand self and current problems, whereas brooding rumination is a form of passive focusing on symptoms and their consequences. The two subtypes of rumination have been considered as distinct coping strategies. More specifically, brooding is considered as a maladaptive coping strategy, whereas reflection is viewed as less problematic as a coping strategy. Most studies examining the effects of rumination subtypes on depression have found concurrent associations with depression symptoms. A meta-analysis of cross-sectional studies indicates that brooding yields a stronger association with depression symptoms than reflection (*ρ* = .61 and *ρ* = .41, respectively; [20]); however, longitudinal analyses indicate differential association with depression for each subtype. For example, over time brooding may be associated with increased depression symptoms, whereas reflection may be associated with fewer depression symptoms [21–23]. Marroquín and colleagues [24] evidenced brooding as associated with passive coping and subsequently linked to depression, whereas reflection was more strongly associated with active coping. The adaptive effects of reflection via the active coping pathway may be dependent on the context of all coping strategies that are available for use among individuals [24,25]. Brooding, but not reflection, consistently mediates the relationship between vulnerability factors and depressive symptoms (e.g., [23,24,26–28]). The available results suggest that brooding and reflection may exert differential effects on depression, wherein brooding is more related to the negative outcomes of rumination and the risk of the development and maintenance of depressive symptoms [22].

### Intolerance of uncertainty and depression

Ruminative styles in depression may represent a tendency to collect or consolidate information in response to real or perceived uncertainty. Specifically, rumination may be a specific cognitive by-product of IU. Carleton [29] defined IU as “an individual’s dispositional incapacity to endure the aversive response triggered by the perceived absence of salient, key, or sufficient information, and sustained by the associated perception of uncertainty” (p. 31). IU has typically been examined in the context of anxiety, with compelling evidence of the transdiagnostic and fundamental nature of this construct in anxiety [29–31]; however, there have been relatively fewer investigations focused on IU in the context of depression and depressive symptoms. Individuals with heightened symptoms of depression, and those who meet the diagnostic threshold for depression report significantly higher IU compared to individuals without depressive symptoms [32,33]. Given the consistent link with depression, several researchers have suggested that the transdiagnostic properties of IU extend not only to anxiety, but also to depressive disorders [34,35].

IU may interact with existing vulnerability in depression in several ways. First, IU may act as a moderating variable for rumination, which may explain heightened IU among persons with depression symptoms. Watkins and Baracaia [36] found that many patients with depression perceive rumination as beneficial for understanding, and perhaps ameliorating, their symptoms. Paradoxically, rumination appears to inhibit effective problem solving and emotion regulation through increasing uncertainty [37]. Indeed, rumination, IU, and depressive symptoms appear significantly interrelated among dysphoric (those showing elevated symptoms of depression not reaching clinical threshold) and non-dysphoric participants [38].

Liao and Wei [39] examined whether rumination mediates or moderates the relationship of IU and depression symptoms in a sample of 332 undergraduate students using a cross-sectional design. The authors hypothesized that the relationship between IU and depression symptoms varies depending on rumination levels, such that higher levels of rumination would exacerbate the association between IU and depression. The authors also hypothesized an indirect effect of rumination in the relationship between IU and depression. Their results supported both hypotheses; specifically, rumination fully mediated the relationship of IU and depression symptoms. The researchers also found the relationship of depression and IU was moderated by rumination such that the relationship between IU and depression intensified among participants reporting heightened rumination [39]. The mediational properties of rumination in the relationship between IU and depression were also found among a clinical sample [40].

The available evidence suggests: 1) rumination is consistently associated with depression symptoms, and is a maladaptive strategy to regulate negative emotions; 2) IU may be a transdiagnostic construct, associated with both heightened depression and anxiety symptoms; and 3) IU may be linked with depression symptoms through interactive properties with rumination, which may mediate and moderate the IU-depression association. Most studies to date have used cross-sectional designs to establish the association between IU, rumination, and depressive symptoms, prohibiting determinations of temporal precedence. Rumination subtypes are distinct coping strategies that have differential effects on depression symptoms (e.g., [20]), suggesting the mediating and moderating role of rumination may be attributable to brooding rather than reflection; however, there is no empirical data demonstrating the specific effects of rumination subtypes on IU and depression.

### Current study

The current study was designed to replicate and extend previous studies demonstrating the mediation and moderation effects of rumination in the relationship of IU and depression symptoms among general community participants using both cross-sectional and longitudinal designs. In Study 1, we employed a cross-sectional design to replicate previous studies examining the putative effects of IU and rumination in depression. Study 2 was designed to replicate results obtained in Study 1 using a longitudinal design. Data from Study 2 would also allow assessing indirect and moderation effects of brooding and reflection on the relationship between IU and depression. Consistent with previous studies [33,38,39], we hypothesized that (1) rumination would mediate the relationship of intolerance of uncertainty and depression symptoms, and (2) rumination would moderate the relationship between intolerance of uncertainty and depression symptoms.

## Study 1

### Method

#### Participants and procedure

There were 572 participants recruited from the community via CrowdFlower, an international online crowdsourcing platform. Web-based crowdsourcing platforms are becoming popular among social scientists and clinical researchers for scientific survey research [41,42]. There were 78 out of 572 individuals with 10% or more of incomplete data were removed from subsequent analyses, resulting in a final sample size of 494 participants. Only 487 participants reported their gender, of which 42.1% (*n* = 205) were men, 56.9% (*n* = 277) were women, and 1.0% (*n* = 5) identified as gender neutral. Table 1 provides detailed demographic characteristics data.

**Table 1:** Demographic Characteristics of the Overall Sample (n = 494).

Age (*SD*)	37.10 (12.33)
Gender (%)	
Women	56.60
Men	42.40
Gender Neutral	1.00
Ethnicity (%)	
White	86.00
Other	14.00
Marital Status (%)	
Single	46.90
Married	43.60
Separated/Divorced	8.10
Widowed	1.40
Annual Income (%)	
< $30,000	41.50
$30,001–$50,000	23.10
> $50,000	35.30
Education (%)	
Secondary/below	24.20
Trades certificate/diploma	29.50
Bachelor’s degree	30.40
Above bachelor’s	15.90

All of the study materials (e.g., consent form, demographics form, questionnaires, and debriefing form) were administered electronically via Qualtrics, an internet-based survey and data collection platform, and disseminated to participants through CrowdFlower. The presentation order of individual questionnaires was randomized across participants. Study 1 was approved by the University of Regina Research Ethics Board (REB#: 2015–203).

### Measures

#### Patient health questionnaire-9 (PHQ-9; [43])

The PHQ-9 is a 9-item self-report questionnaire designed to assess depression, in accordance with the nine diagnostic criteria of major depression in the Diagnostic and Statistical Manual of Mental Disorders-4 (DSM-IV; [44]). Each item is rated on a 4-point Likert scale ranging from 0 (*not at all*) to 3 (*nearly everyday*). Scores on the PHQ-9 demonstrated good psychometric properties among clinical and nonclinical populations (e.g., [45,46]). The scale has also been shown to have strong convergent validity with other depression measures, such as the Beck Depression Inventory-II (BDI-II; [47]) and the Center for Epidemiological Studies—Depression Scale (CES-D; [48]), with correlations ranging from .67 to .81 for the BDI-II [49] and .72 to .84 for CES-D (e.g., [50]). The current study used eight items (PHQ-8) from the original measure after the removal of the suicide item. Cronbach’s alpha in the current sample was .89.

#### Ruminative response scale (RRS; [51])

The RRS is a 22-item self-report measure used to assess trait rumination—or the tendency to which the individual focuses on the causes, consequences, and symptoms of a depressed mood. The RRS consists of three subscales: brooding, reflection, and depression-related rumination. The 22 items are rated on a 4-point Likert scale ranging from 1 (*strong disagree*) to 4 (*strongly agree*). Scores can range between 22 and 88, with higher scores indicating higher tendency to ruminate. The RRS has demonstrated good internal consistency, test-retest reliability, and validity for predicting depression [7,52,53]. The scale also demonstrated good to excellent internal consistency in the current sample, with Cronbach’s alphas value of .94 for the total scores, as well as .81 and .78 for the brooding and reflection subscales, respectively.

#### Intolerance of uncertainty scale, short form (IUS-12; [54])

The IUS-12 was derived from the original 27-item Intolerance of Uncertainty Scale [55] which measures reactions to ambiguous situations, uncertainty, and the future (e.g., “*I always want to know what the future has in store for me”)*. Each item is rated on a 5-point Likert scale from 1 (*not at all characteristics of me*) to 5 (*entirely characteristic of me*), with possible scores ranging between 12 and 60. The IUS-12 has demonstrated good convergent and discriminant validity [33,54], strong correlations with other measures of the same construct, and strong correlations with original 27-item scale in both undergraduate and clinical samples [33,54,56]. Cronbach’s alpha in the current study was .90.

#### Statistical analyses

Moderation and mediation analyses were conducted using the bootstrapping technique. The bootstrapping moderation analysis was performed using the PROCESS macro for SPSS [57] to assess the moderating role of rumination in the relationship between IU and depression (see S1 Dataset). The PROCESS macro determines centering and interaction terms, as well as providing the point estimate, and first- and second-order variance estimate of the conditional effect for a given set of moderator values. The Liao and Wei [39] results were extended by conducting two additional moderation analyses to assess the interactive effects between IU and the unique subcomponents of rumination on depression; specifically, the two RRS subscale scores were entered independently as moderators.

To examine the mediating role of rumination in the relationship of IU and depression, bootstrapping mediation analyses were also conducted to assess the mediating role of rumination in the relationship of IU and depression [58]. Based on the distribution of the resampled dataset, 95% CIs were generated for the total, direct, and indirect effects being examined [57,59]. The Liao and Wei [39] results were further extended by delineating the effect of different components of rumination on the relationship between IU and depression; specifically, three additional independent mediation analyses were conducted using the subscale scores of the RRS as mediators: brooding and reflection.

### Results

#### Moderation analysis

Using model 1 in the PROCESS macro for SPSS, the analysis indicated the overall model was significant, *R*^*2*^ = .47, *F*(3, 484) = 138.37, *p* < .001; however, the interaction between IU and rumination was not significant (IU x rumination: *b* = .002, *t* = 1.58, *p* = .12). Accordingly, rumination did not appear to significantly moderate the relationship between IU and depression. There were also no statistically significant interaction effects found between IU and each component of rumination: brooding (*p* = .44) and reflection (*p* = .74).

#### Mediation analysis

**Fig 1:**
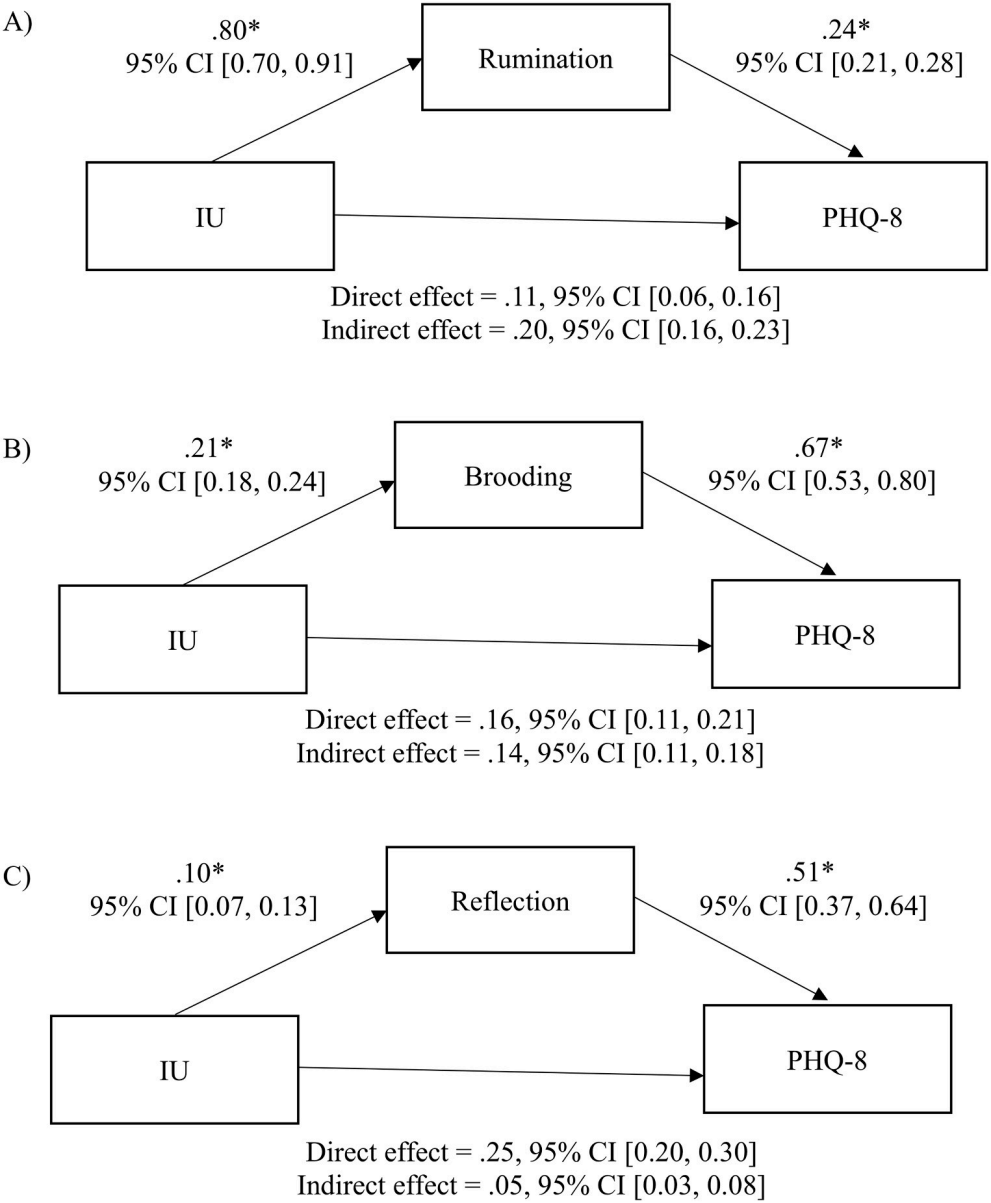
Cross-sectional Mediation models. (A) The indirect effect of total rumination scores on the relationship between intolerance of uncertainty (IU) and depression, *R*^*2*^ = .46, *F*(2, 485) = 208.41, *p* < .001. (B) The indirect effect of brooding scores on the relationship between IU and depression, *R*^*2*^ = .37, *F*(2, 485) = 139.16. (C) The indirect effect of reflection on the relationship between IU and depression, *R*^*2*^ = .032, *F*(2, 485) = 154.34, *p* < .001. *p < .001.

Using model 4 in the PROCESS macro for SPSS, a significant overall model was found between IU, rumination and depression, *R*^*2*^ = .24, *F*(2, 486) = 208.41, *p* < .001. There was a significantly partial indirect effect for rumination in the relationship between IU and depression (*b* = .20, 95% BCa CI [.16, .23]). See Fig 1A.

There were two additional mediation analyses were conducted using the brooding and reflection subscales to delineate effects on the relationship between IU and depression. Additional analyses indicated that both subscales yielded significant partial indirect effects. Specifically, a small-to-medium mediating effect was found for brooding (*b* = .14, 95% BCa CI [.11, .18), and a small effect was found for reflection (*b* = .05, 95% BCa CI [.03, .08]). See Fig 1 for additional mediation models.

## Study 2

### Methods

#### Participants & procedure

**Fig 2:**
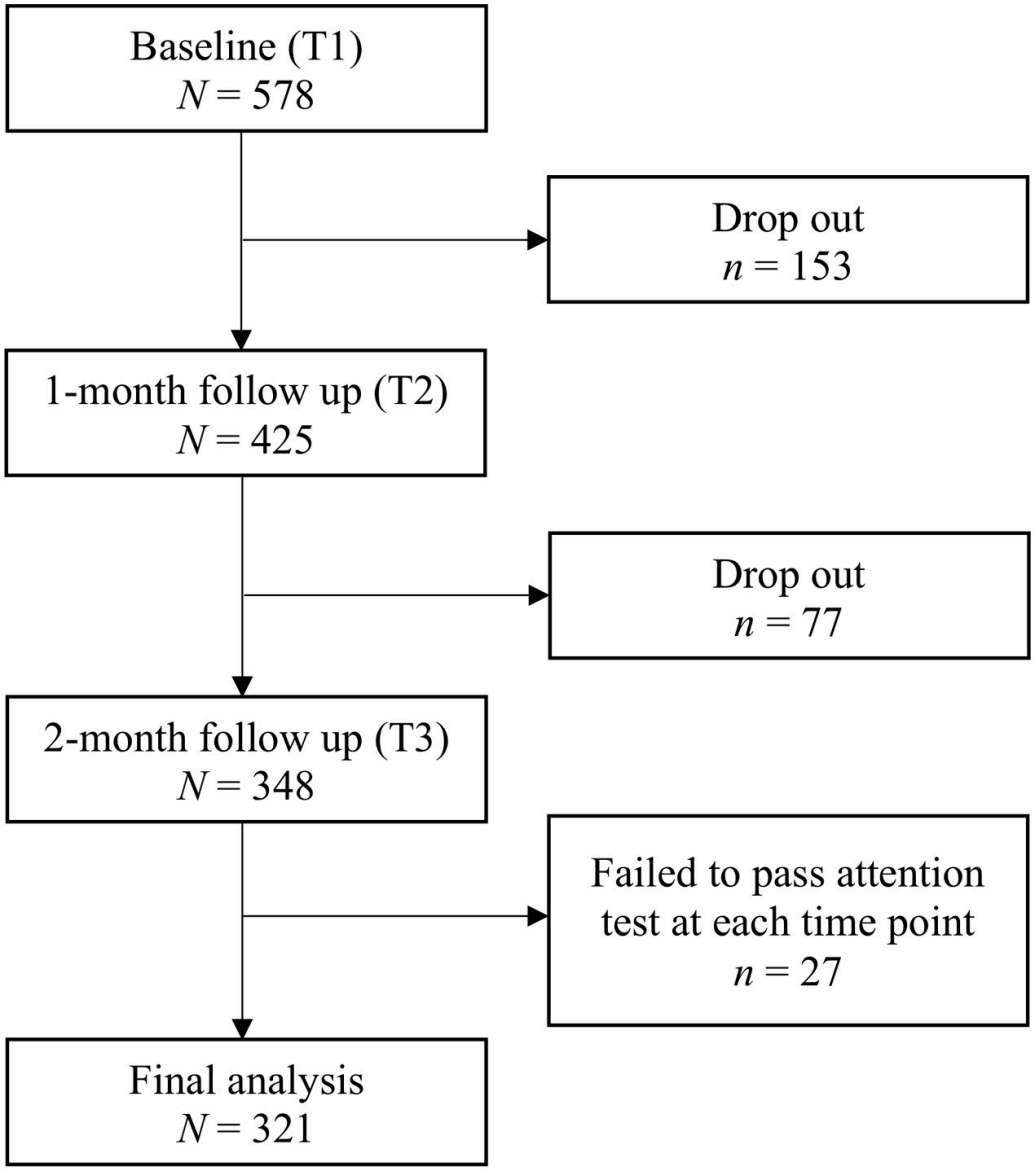
Sample retention of Study 2.

A total of 578 participants were recruited at baseline from the community via Amazon’s Mechanical Turk, an US-based online crowdsourcing platform. There were 425 out of 578 baseline (T1) participants who completed the 1-month follow-up (T2) and 348 who completed the 2-month follow-up (T3). Fig 2 contains sample retention details. There were significant differences between completers and non-completers at T2 and T3 for age, marital status, and baseline PHQ-8 and RRS total scores (*ps* < .05). Descriptive statistics between completers and non-completers are presented in Table 2. Specifically, participants who dropped out at T2 were younger and more likely to be women than completers. Participants who dropped out at T3 were more likely to be single and had higher baseline PHQ-8 and RRS total scores than completers, and higher than participants who dropped out at T2.

**Table 2:** Descriptive statistics of demographic and study variables between completers and non-completers.

	Completers *n* = 341	Non-completers from T1 to T2 *n* = 129	Non-completers from T2 to T3 *n* = 78	*p*
Age (*SD*)	33.83 (11.84)	33.19 (9.22)	33.74 (10.98)	**.002**
Men (%)	51.6	41.9	46.2	.52
Marital status (%)				**.005**
Single	57.2	51.2	69.2	
Married	32.0	45.0	20.5	
Separated/Divorced	9.7	3.9	10.3	
Widowed	1.2	0	0	
Education (%)				.81
Below high school	1.2	0.8	0	
High school or equivalent	20.2	25.6	20.5	
Bachelor’s degree	19.4	19.4	23.1	
Above Bachelor’s	48.7	45.0	42.3	
Other	10.6	9.3	14.1	
Income Level (%)				.28
Under $30,000	42.8	34.1	39.7	
$30,001–$50,000	28.2	31.0	35.9	
Over $50,000	29.0	34.9	24.4	
Past History of Depression (%)	56.9	58.1	62.8	.60
PHQ-8 total score, T1	6.84 (6.46)	7.91 (6.63)	9.06 (7.42)	**.02**
IUS total score, T1	33.37 (11.47)	32.74 (10.33)	34.55 (12.61)	.53
RRS total score, T1	44.19 (14.84)	46.39 (15.57)	49.24 (15.58)	**.02**

*Notes*. IUS = Intolerance of Uncertain Scale; PHQ-8 = Patient Health Questionnaire– 8 Items; RRS = Ruminative Response Scale; T1 = Baseline measure; T2 = 1-month follow-up; T3 = 2-month follow up.

Paralleling the cross-sectional design, all study materials were administered electronically via Qualtrics. The same set of questionnaires were randomized across participants at each time point. Attention check questions were administered at each time point to ascertain effort and attention during questionnaire completion. Participants were then thanked and debriefed upon completion at T3. All participants were financially compensated in line with community expectations for MechanicalTurk. Only participants who passed the attention check at all three time points were included in the final analysis, which resulted in a final sample size of 321 participants. The sample mean age was 36.76 (*SD* = 11.71), half of the participants were men (51.7%), and most participants self-identified as White (77.3%). Demographic details are presented in Table 2. Study 2 was approved by Ryerson University Research Ethics Board (REB#: 2019–192).

#### Measures

Study 2 employed the same measures as Study 1 to assess depression (PHQ-9), IU (IUS), and rumination (RRS). In the present study, each scale yielded good to excellent internal consistencies at each time point. For the PHQ-9, the Cronbach’s alphas were .91 at T1, .92 at T2, and .91 at T3. The Cronbach’s alphas for the IUS were .94 at all three time points. For the RRS, the Cronbach’s alphas for the total score, as well as the brooding and reflection subscale scores were, respectively, .95, .80, and .86 at T1, .96, .88, and .85 at T2, and .97, .88, and .85 at T3.

#### Statistical analysis

Moderation analysis was conducted using a multilevel modeling (MLM) approach. The MLM approach is more appropriate for hierarchically nested data structures such that a lower level unit of analysis (level 1; i.e., repeated measures of the three constructs) is nested within a higher level of analysis (level 2; e.g., the participant). Specifically, three random-intercepts models with a restricted maximum likelihood estimator were used to assess the interaction between IU and rumination (i.e., brooding, reflection and total scores) on depressive symptoms over time. These analyses were conducted using R [60] via the “nlme” (Linear and Nonlinear Mixed Effects Models) [61] and “interaction” [62] statistical packages.

Mediation analysis was conducted using bootstrapping method via the PROCESS macros on SPSS (model 4) [57]. In order to establish the temporal precedence between the three constructs, three mediation models adjusting for the baseline PHQ-8 total score were conducted. Specifically, the baseline (T1) IUS total score was entered as the independent variable, the RRS total scores, and brooding and reflection subscale scores at T2 were each entered as the mediator, and the PHQ-8 total score at T3 was entered as the dependent variable (S1 Dataset).

### Results

#### Moderation analysis

The MLM analyses indicated that there was no statistically significant main effect of time, nor a statistically significant interactive effect of IUS x RRS x time on depressive symptoms. Similarly, no statistically significant main effects of time and interactive effects of IUS x Brooding x time, or IUS x Reflection x time were associated with depressive symptoms in the other two models (see Table 3 for detailed results).

**Table 3:** The Moderating Effects of IU and Rumination on Depression over Time.

*RRS total score*		
Fixed effects	Coefficient	*p* [95% CI]
Intercept	0.63	.77 [-3.60, 4.85]
IUS	-0.08	.22 [-0.20, 0.05]
RRS	0.05	.35 [-0.05, 0.14]
Time	-0.70	.40 [-2.35, 0.95]
IUS x Time	0.03	.29 [-0.02, 0.08]
IUS x RRS	< 0.01	.001 [0.002, 0.007]
RRS x Time	0.03	.16 [-0.01, 0.07]
IUS x RRS x Time	< -0.01	.08 [-0.002, 0.0001]
Random effects	Variance	
σ^2^	5.16	
τ	13.31	
*RRS Brooding*		
Fixed effects	Coefficient	*p* [95% CI]
Intercept	1.30	.50 [-2.50, 5.09]
IUS	0.01	.86 [-0.10, 0.12]
Brooding	0.03	.88 [-0.35, 0.41]
Time	-0.60	.43 [-2.07, 0.88]
IUS x Time	0.02	.46 [-0.03, 0.06]
IUS x Brooding	0.01	.008 [0.003, 0.02]
Brooding x Time	0.12	.11 [-0.03, 0.28]
IUS x Brooding x Time	-0.003	.09 [-0.007, 0.001]
Random effects	Variance	
σ^2^	5.34	
τ	18.44	
*RRS Reflection*		
Fixed effects	Coefficient	*p* [95% CI]
Intercept	2.98	.17 [-1.32, 7.29]
IUS	0.02	.81 [-0.11, 0.14]
Reflection	-0.23	.28 [-0.66, 0.19]
Time	-0.49	.56 [-2.14, 1.16]
IUS x Time	0.01	.65 [-0.04, 0.06]
IUS x Reflection	0.02	.005 [0.005, 0.03]
Reflection x Time	0.10	.23 [-0.06, 0.27]
IUS x Reflection x Time	< -0.1	.22 [-0.007, 0.002]
Random effects	Variance	
σ^2^	5.58	
τ	20.95	

#### Mediation analysis

**Fig 3:**
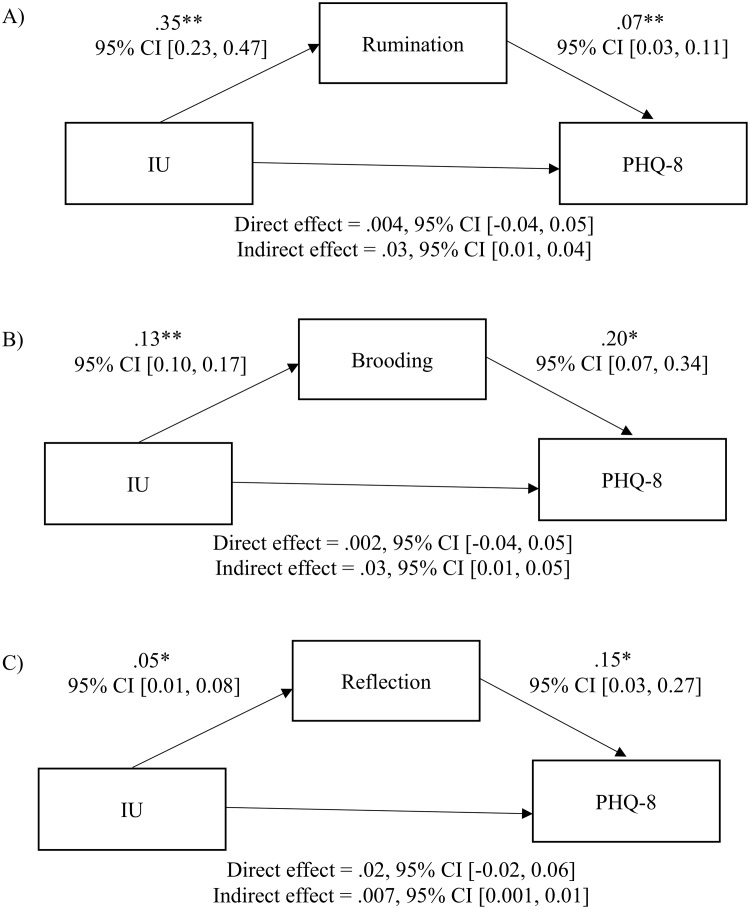
Longitudinal mediation models. (A) The indirect effect of total rumination scores on the relationship between IU and depression, *R*^*2*^ = .69, *F*(3, 317) = 235.72, *p* < .001. (B) The indirect effect of brooding on the relationship between IU and depression, *R*^*2*^ = .69, *F*(3, 317) = 230.05, *p* < .001. (C) The indirect effect of reflection on the association between IU and depression, *R*^*2*^ = .68, *F*(3, 317) = 226.29, *p* < .001. * *p* < .01, ** *p* < .001.

After controlling for PHQ-8 total scores at T1, results from the mediation analyses indicated statistically significant indirect effects of total scores on rumination scale (RRS), but also subscale brooding and reflection scores on the relationship between IU and depression (see Fig 3 for detailed results). Specifically, the magnitude of the indirect effects of brooding and the total RRS scores were comparable, but the magnitude of indirect effect was larger for brooding than that of reflection.

## Discussion

IU, and in particular rumination, have been implicated in depression with evidence from several studies; however, very few studies to date have examined the interactive effects of IU and rumination factors in the development and maintenance of depression. Importantly, no studies to date assessed for temporal precedence of IU and rumination in their maintenance of depression. The current study was designed to replicate and extend existing literature on the interplay between IU, rumination, and depression by using both a cross-sectional (Study 1) and longitudinal (Study 2) designs. There is cross-sectional support for the indirect association of IU and depression through rumination [39, 40]; however, cross-sectional mediation models are necessarily limited particularly when assessing mediation [63–65]. The current study is the first longitudinal study assessing the relationship and clarifies the role of IU as a vulnerability factor for depression symptoms through the established pernicious mechanism of heightened rumination. The current results support the need for innovating interventions specifically designed to disrupt the pernicious and reflexive process of IU and rumination in depression.

Rumination mediated the relationship between IU and depression in both Study 1 (cross-sectionally) and Study 2 (longitudinally). The brooding and reflection rumination subtypes both mediated the association between IU and depression; however, brooding yielded a stronger indirect effect on the IU-depression relationship compared to reflection. There were minor differences in the estimates of the mediational effect across both our studies; specifically, rumination only partially mediated the IU-depression relationship in Study 1, whereas full mediation was found in all three models in Study 2. The current results replicated previous cross-sectional results [39, 40] and provide the first evidence of temporal precedence; that is, high levels of IU appeared to support the development of depression symptoms over time through the engagement in heightened rumination. The longitudinal results are consistent with the notion that rumination, and especially brooding, is a maladaptive coping strategy that intensifies the relationship between cognitive vulnerability factors and the associated negative psychological outcomes. Individuals who are intolerant of uncertainty may lack sufficient problem-solving skills, which in turn is associated with higher levels of distress [40]. Spasojević and Alloy [52] indicated that individuals with cognitive vulnerabilities for depression, such as IU, tended to engage in rumination to cope with negative emotions associated with perceived uncertainty [36,66]. Rumination may intensify negative emotions associated with uncertain situations rather than reduce the ability to engage in problem-solving strategies [5,39], increasing vulnerability to depressive symptoms [66].

Results from the current study contrast previous cross-sectional results [24,39,40] by evidencing reflection as mediating the relationship between IU and depression; however, the mediating effect was larger for brooding than for reflection. Brooding is a passive coping strategy associated with poor decision making, less effective problem solving, and higher depression symptoms over time (e.g., [9,36,67]); in contrast, reflection is conceptualized as an active coping strategy [24,68] associated with lower depression symptoms over time [24,53]. The relative benefits of reflection may be context dependent [25] or mitigated by IU which negatively biases attention and information processing of ambiguous events [69]. IU may facilitate negative thoughts, providing opportunities for individuals engaging in reflection to elaborate on negative cognitions, further facilitating heightened depressogenic beliefs. Overall, it appears that the association between IU and depression may be explained through subcomponents of rumination. Additional longitudinal research is needed to delineate the interplay between components of rumination, IU, and depression.

The moderating role of rumination, including brooding and reflection, in the relationship between IU and depression was not supported in both the cross-sectional and longitudinal designs. The current results contrasted our hypothesis and results from previous findings [39]. The non-significant moderation effects could be attributed to methodological differences with previous study research. For example, Liao and Wei [39] found evidence of a moderation effect using an undergraduate sample, but combined the standardized scores on two different scales to assess rumination instead of using only the RRS. Liao and Wei [39] also assessed depression symptom frequency and severity using multiple self-report scales instead of using only the PHQ-8. Future studies should use more comprehensive measures to assess each of the constructs to delineate directionality between the variables examined, especially in clinical samples.

The current study has several limitations that offer directions for future research. First, the use of an online community sample did not allow the examination of the variables of interest in the context of clinical diagnoses. As such, the present study design relied on the use of self-report measures to assess depressive symptoms, and thus assessment of diagnostic threshold based on self-reports may lack the specificity and sensitivity when compared with diagnoses derived from “gold standard” clinical interviews. Future studies should consider using a standard diagnostic clinical interview alongside more diverse self-report depression measures to better evaluate depressive symptoms and diagnoses. Second, the present study utilized crowdsourcing recruitment techniques to obtain the sample, which may have introduced potential sampling biases, and thus the sample may not be fully representative of the general population [41]. Thus, the present sampling method might hinder the generalizability of the current findings. Third, no attention or validity check was embedded into Study 1. As such, findings from Study 1 should be interpreted with caution. Such methodology limitation was rectified in Study 2 in which attention check items were embedded at all three time points in order to identify random responding. Lastly, future studies should employ a longitudinal design with longer durations to provide a better understanding of the stability of the mechanism that underlies rumination and its subtypes, IU, and depression.

Despite the aforementioned limitations, the presented study extends previous work in a several ways. First, the current study is the first to examine the interplay (e.g., moderation and mediation) between IU, rumination, and depression using a longitudinal design. Second, although rumination is a multi-faceted variable, researchers have often examined the role of global rumination in relation to depression (i.e., using total rumination scores), neglecting the importance and potential differences effects of its subtypes. The current study extended previous research by assessing brooding and reflection rumination subtypes using mediation and moderation models, and evidencing different pathways for relationships between IU, rumination, and depression. Overall, the results support the potential of treatments targeting IU for depression to influence symptoms through diverse pathways.

## Supporting information

S1 Dataset (ZIP)
